# Know Your ABCs: Discovery, Differentiation, and Targeting of T‐Bet+ B Cells

**DOI:** 10.1111/imr.13440

**Published:** 2025-01-22

**Authors:** Gary M. Winslow, Russell Levack

**Affiliations:** ^1^ Department of Microbiology and Immunology Upstate Medical University Syracuse New York USA; ^2^ Department of Immunology University of Pittsburgh Pittsburgh Pennsylvania USA

**Keywords:** ABCs, adenosine receptor, age‐associated B cells, CD11c, ehrlichia, T‐bet B cells

## Abstract

Since their first description in 2008, T‐bet+ B cells have emerged as a clinically important B cell subset. Now commonly known as ABCs (Age‐associated B Cells), they are uniquely characterized by their expression of the transcription factor T‐bet. Indeed, this singular factor defines this B cell subset. This review will describe the discovery of T‐bet+ B cells, their role in bacterial infection as T cell‐independent (TI) plasmablasts, as well as long‐term follicular helper T cell‐dependent (TD) IgM+ and switched memory cells (i.e., T‐bet+ ABCs), and later discoveries of their role(s) in diverse immunological responses. These studies highlight a critical, although limited, role of T‐bet in IgG2a class switching, a function central to the cells' role in immunity and autoimmunity. Given their association with autoimmunity, pharmacological targeting is an attractive strategy for reducing or eliminating the B cells. T‐bet+ ABCs express a number of characteristic cell surface markers, including CD11c, CD11b, CD73, and the adenosine 2a receptor (A2aR). Accordingly, A2aR agonist administration effectively targeted T‐bet+ ABCs in vivo. Moreover, agonist treatment of lupus‐prone mice reduced autoantibodies and disease symptoms. This latter work highlights the potential therapeutic use of adenosine agonists for treating autoimmune diseases involving T‐bet+ ABCs.

## 1 The Discovery of T‐Bet+ B Cells

T‐bet+ B cells were first discovered as part of our studies of a bacterial infection in mice [1]. The B cells were first characterized based on their expression of CD11c, a cell surface marker that had been well‐known to be characteristic of dendritic cells, and others. Although there had been reports of CD11c expression in B cells, primarily from molecular studies [2], and in a report of a hairy cell leukemia [3], our studies were the first to identify CD11c + B cells in vivo [1]. We did not identify T‐bet expression in the CD11c + B cells in our initial studies. However, it is clear in hindsight, based on their phenotype, that the cells were indeed what are now known as T‐bet+ B cells [4]. T‐bet expression was first reported in B cells in a study that identified T‐bet as an important transcription factor that drives the differentiation of CD4 T cells [5]. However, no further studies of T‐bet‐expressing B cells were reported until T‐bet was identified as a lineage‐defining transcription factor in a B cell subset in mice and aged autoimmune humans [6, 7]. In these later studies, the B cells were named ABCs [6]. Classification of these cells has been confounded by the heterogeneity of T‐bet‐expressing B cell populations. For example, *E. muris* infection elicits a population of T‐bet+ extrafollicular plasmablasts as well as a temporally distinct population of T‐bet+ memory B cells; the latter more closely resemble canonical ABCs. Here, we have adopted the term “T‐bet+ ABCs” to describe the memory B cells we originally identified, to differentiate them from other T‐bet+ B cells. Although T‐bet+ ABCs express a number of signature cell surface markers (see below), they are phenotypically diverse. For example, the cells were originally identified on the basis of their unexpected expression of CD11c, but in later studies CD11c‐negative T‐bet+ ABCs were also identified [1, 8]. However, a common essential feature of T‐bet+ ABC function in diverse contexts is the production of IgG2a/c, as will be discussed below [9].

Since their initial discovery, and following their identification in aging and autoimmunity, T‐bet expression has been documented in our studies in several B cell populations, including IgM‐producing TI extrafollicular plasmablasts, and IgM and switched memory cells. T‐bet‐expressing B cells have also been found in anti‐viral and bacterial immune responses, and in a range of autoimmune diseases, including lupus, multiple sclerosis (MS), Sjøgren's Syndrome, rheumatoid arthritis (RA), obesity‐related autoimmunity, and others [7, 10, 11, 12, 13, 14, 15, 16, 17, 18].

As our studies were performed primarily in a bacterial infection, this review will focus on our findings from that model, and the implications of the work for B cell biology as a whole, and for autoimmunity in humans. We will also describe the role of T‐bet+ ABCs in autoimmune diseases, including systemic lupus erythematosus (SLE), and report on studies that have successfully targeted the cells in vivo in lupus‐susceptible mice. The latter work provides a means to treat autoimmune diseases whose etiology is partly or wholly dependent on T‐bet+ ABCs.

### 1.1 The Ehrlichial Infection Model

Our studies used a mouse model to study immunity to the ehrlichiae. Ehrlichiae are a group of tick‐transmitted, gram‐negative Rickettsiae known to cause several related diseases in humans and animals, such as human monocytotropic ehrlichiosis (HME) [19]. HME is a zoonotic disease transmitted by the Lone Star Tick. Our initial studies utilized *E. chaffeensis* , the causative agent of HME. However, *E. chaffeensis* infection in immunocompetent mice is not robust, causing at most modest disease. Thereafter, we focused on a related ehrlichia, *E. muris* , and in some cases, *Ixodes ovatus* ehrlichia (IOE); the latter pathogen causes fatal infection in naive laboratory mice. However, the lack of an effective immune response against the highly pathogenic IOE made it impossible to study protective immunity in the absence of prior immunization.

The ehrlichiae are monocytotropic, and reside in early endosomes [20]. They infect monocytes/macrophages in all tissues that have been examined [21, 22]. Importantly, in the mouse, infection causes massive changes in spleen architecture and bone marrow hematopoiesis [1, 23]. Splenomegaly, which reaches a maximum on day 18 postinfection, is characteristic of *E. muris* infection in mice. This paucity of lymphoid architecture that typically accompanies a conventional immune response may be responsible for the massive early TI T‐bet+ B cell response in *E. muris* (as many as 10^8^ early plasmablasts are generated per spleen) [1]. In addition to the major changes in lymphoid architecture, infection also causes a major inflammatory response, especially the production of IFNγ, that likely facilitates the development of T‐bet+ plasmablasts and T‐bet+ ABCs [24, 25]. Paradoxically, ablation of the inflammatory cytokine TNF resulted in increased production of T‐bet+ ABCs [26]. The role of inflammatory cytokines in the generation of T‐bet+ ABCs is, therefore, unresolved.

### 1.2 The Importance of Antibodies in Immunity to Intracellular Ehrlichia Infection

The first hint that antibodies were highly effective against the ehrlichiae came from studies of *E. chaffeensis* infection of SCID mice (SCID mice, but not immunocompetent mice, are highly susceptible to fatal *E. chaffeensis* infection). Transfer of immune serum from previously infected immunocompetent mice protected SCID mice from fatal infection, even when antibodies were administered well after infection had been established [27, 28]. These data were enigmatic, given that the ehrlichiae are monocytotropic, and were thought to reside only inside cells, unreachable by antibodies. Although the mechanism whereby antibodies protect highly infected mice has never been fully resolved, at least some “intracellular” ehrlichiae can be found outside of host cells, where they are presumably susceptible to opsonization by antibodies [29]. Indeed, it seems self‐evident that at some point in the bacterial lifecycle, the bacteria gain access to the bloodstream to facilitate blood‐borne transmission by the tick vector. However, the kinetics of bacterial clearance, in as few as 24 h, suggested that additional mechanisms are likely at work.

To better understand the humoral immune response to the ehrlichiae, we characterized a large panel of monoclonal antibodies generated during *E. chaffeensis* infection of immunocompetent C57BL/6 mice. Most monoclonal antibodies were directed at a single epitope in a major ehrlichial outer membrane protein‐19 (OMP‐19) [30], and these antibodies protected SCID mice from infection and disease when administered intraperitoneally, either prior to or after infection [27]. The monoclonal antibodies were predominantly IgG2a and IgG3 (IgM monoclonal antibodies were not studied), and the IgG2a were of exceptionally high affinity [31]. These studies demonstrated that antibodies could be highly effective against intracellular ehrlichiae, and likely other intracellular bacteria. Although the cells responsible for antibody production were not known, it was thought that we had characterized a canonical B cell response, similar to what is generated following most immunizations. Nevertheless, these studies, and others, revealed that antibodies and B cells could play significant roles in immunity in ehrlichia‐infected mice, and led to further studies of humoral immunity to ehrlichial infection.

### 1.3 CD4 T Cell‐Independent Immunity to *E. muris* Infection

In later studies, we transitioned to an *E. muris* infection model. This was because it had been established that prior *E. muris* infection was highly effective at protecting immunocompetent mice from fatal IOE infection [32], thereby providing an excellent animal model system to study protective immunity. Moreover, the primary immune response to *E. muris* infection in immunocompetent mice is highly effective, as *E. muris* infection is largely resolved by day 30 postinfection [1]. In addition, it was later shown that previously infected mice, or mice immunized with OMP‐19, were fully immune to re‐infection with *E. muris* [33, 34].

Because antibodies, primarily IgM and IgG2a, could protect against ehrlichial infection [27, 35], it seemed likely that B cell antibody production was partially or wholly CD4 T cell‐dependent, as CD4 T cells had long been considered to be essential for an effective immune response to intracellular bacteria. However, immunity to *E. muris* could be achieved in the complete absence of CD4 T cells in otherwise immunocompetent mice [34]. This observation that TI immunity was sufficient for protection was fortuitous, as it coincided with the discovery of CD11c + T‐bet+ B cells.

### 1.4 The Discovery CD11c + (T‐Bet+) B Cells

The discovery of CD11c + B cells stemmed from our exploratory studies of dendritic cell (DC) immunity in *E. muris* ‐infected mice [1]. Infection induces a major population of CD11c + MHC class II+ cells, beginning on about day nine postinfection, when it represents as many as 15% of spleen mononuclear cells. Further characterization revealed that the cells were, in fact, not DCs, but were IgM/IgD+ B cells [1]. Other cell surface markers characteristic of the CD11c + B cells were the integrins CD11b, CD29, and CD49d, the sialoglycoprotein CD43, the chemokine receptor CXCR4, and the ectonucleotidase CD73. Significantly, at least 70% of the cells detected at that time postinfection were also CD138+, which indicated that the B cells were antibody‐secreting plasmablasts. Indeed, the CD11c + B cells were responsible for nearly all of the IgM produced during infection, as well as all of the OMP‐specific IgM produced in the spleen [1]. The CD11c + B cells were generated in the absence of MHC class II expression, indicating that they were likely generated in a CD4 T cell‐independent fashion. These studies, following the demonstration of protective TI immunity against *E. muris* , identified the CD11c + B cells as the cells likely responsible for the protective antibody response against *E. muris* . Although T‐bet expression had not been detected in the CD11c + B cells at that time, the CD11c + T‐bet+ plasmablasts were novel, and the findings presaged the discovery of what are now known as canonical T‐bet+ ABCs.

Remarkably, IgM production in the *E. muris* ‐infected mice continued for at least as long as 400 days postinfection, even after long‐term antibiotic treatment (the ehrlichiae are sensitive to the antibiotic doxycycline) [35]. Many of the long‐term plasmablasts were found and persisted in the bone marrow, which suggested that IgM production could be maintained indefinitely at that site, and was responsible for the long‐term protection against fatal infection [35]. Much of the IgM produced by the T‐bet+ B cells was polyreactive, a property that allows individual IgM to bind to diverse, structurally unrelated, antigens [36]. The significance of this observation is not known, and although relatively novel, it is not thought that the production of polyreactive antibodies is a unique property of IgM‐producing CD11c + T‐bet+ B cells. The spleen CD11c + B cell response, coupled with the loss of spleen cell architecture, was apparently sufficiently robust such that it inhibited antigen‐specific CD4 T cell‐dependent B cell responses to unrelated antigens [37].

This work, in addition to providing the first identification and description of T‐bet+ B cells, revealed that these B cells could function as long‐term antibody‐producing plasmablasts, and plasma cells. Eventually, it would be shown that these are only one of the many functions played by these cells. Indeed, the discovery of the CD11c + plasmablasts was just the beginning of the study of T‐bet+ ABCs.

### 1.5 Switched CD11c + T‐Bet+ Memory ABCs

As was indicated above, the T‐bet+ CD11c + plasmablasts declined in number and frequency in the spleen, once infection was under control by the host [1]. During the course of those studies, however, a second population of CD11c‐expressing B cells was discovered in *E. muris* ‐infected mice. These B cells, which represented approximately 5% of spleen mononuclear cells, were first detected on or about day 30 postinfection in the spleen, but persisted for at least as long as 400 days [38]. T‐bet+ CD11c + B cells were also later found in the peritoneal cavity, which serves as a major reservoir of these cells [39]. The splenic CD11c + B cells were primarily IgM+, and about 50% were also IgD+. The B cells exhibited high surface expression of CD23 and low CD21, indicative of a follicular B cell origin. Still, the B cells did not exhibit a phenotype characteristic of germinal center (GC) B cells. Neither were they antibody‐producing B cells, as evidenced by their lack of CD138 expression. These late‐arising CD11c + B cells also persisted, albeit at somewhat lower frequency, following antibiotic administration, indicating that long‐term maintenance likely was either independent of, or required low level exposure to bacterial antigens ( *E. muris* infection is largely resolved within 30 days postinfection, but may persist indefinitely). Detailed flow cytometric characterization also demonstrated that the CD11c + B cells also exhibited cell surface expression of the proliferation‐inducing glycoprotein CD38, the ectoenzyme CD73, the costimulatory molecule CD80, the proapoptotic receptor CD95, and the inhibitory receptor PD‐L2, all markers that had previously been shown to be expressed by canonical memory B cells [38, 40]. Subsequent studies revealed that the CD11c + B cells also expressed T‐bet [41]. Moreover, bromodeoxyuridine (BrdU) incorporation studies, which have been used commonly to detect cells that had undergone recent cell division, revealed that the B cells were largely quiescent. Moreover, most of the CD11c + B cells, unlike the CD11c + plasmablasts, were generated in a TD fashion, requiring both CD4 and the IL‐21 receptor for their development [38]. The B cells also exhibited B cell receptor somatic mutations, a hallmark of TD antigen‐experienced memory B cells [38]. These findings suggested that the CD11c + (T‐bet+) B cells (T‐bet expression was demonstrated in later studies; see below), were in fact IgM+ memory cells. The findings were remarkable, not only for the high frequency of IgM+ memory cells that had been generated, but because, in mice, memory B cells had long been shown to be maintained in vivo at very low frequency and number, which caused them to be difficult to detect [42, 43, 44, 45]. The identification of large numbers of memory B cells may be attributed, in part, to the robust inflammatory environment generated by *E. muris* infection. Regardless, the studies demonstrated that bacterial infection can generate large populations of long‐term memory B cells. In addition to the relevance of the findings to T‐bet+ ABCs, they were also important for demonstrating that B cell memory could be maintained in nonswitched memory B cells, not only isotype‐switched B cells.

Formal demonstration that the CD11c + (T‐bet+) B cells (described here as T‐bet+ ABCs) were responsible for immunological memory came from experiments that eliminated the CD11c + B cells in vivo, using inducible cell type‐specific bone marrow chimeric gene‐targeted mice [38]. These mice expressed the simian diphtheria toxin receptor (DTR) under the regulation of CD11c (allowing the B cells to be ablated, using diphtheria toxin; DT). This approach was highly effective at targeting CD11c + B cells, without significantly impacting DC function [38]. To address the role of the CD11c + B cells in amnestic responses, the putative memory cells were allowed to accumulate for at least 30 days postinfection, at which time treated and control mice were administered DT, followed by administration of recombinant OMP‐19 antigen 6 days later. The use of OMP‐19 facilitated the analysis of antigen‐specific B cells in the absence of secondary infection (C57BL/6 mice are completely resistant to secondary infection due to the presence of pre‐existing antibodies, which presumably act by preventing bacterial entry into host cells). Serum was harvested 6 and 12 days following antigen administration, and OMP‐19 IgG titers were determined. Remarkably, the secondary IgG response to OMP‐19 was ablated in the mice that no longer carried CD11c + memory B cells [38]. These studies formally demonstrated that the CD11c + (T‐bet+) B cells (i.e., T‐bet+ ABCs) can function as memory B cells. Although the CD11c + cells were largely IgM‐positive, it was concluded that the reason for the reduction of IgG2c following DT administration was because the IgM+ memory cells had undergone class switching following OMP‐19 antigen challenge, as is well‐known to occur in classical models of secondary B cell responses.

The observation that many of the T‐bet+ ABCs had introduced somatic mutations indicated that the B cells at some time in their development had expressed Activation‐Induced Cytidine Deaminase (AICDA), the enzyme responsible for somatic B cell receptor mutations. In later studies, this approach was used to target and identify AIDCA‐expressing memory B cells. This was accomplished using AID‐regulated tamoxifen‐inducible Cre‐recombinase enhanced Yellow Fluorescent Protein (eYFP) reporter mice (AID‐Cre‐ER^T2^ × Rosa26 eYFP F1 mice). B cells in these mice that expressed *Aicda* during the time of tamoxifen exposure were irreversibly marked by EYFP expression [46]. In this manner, memory cells and bone marrow antibody‐secreting cells (ASCs) were marked when tamoxifen was administered on day 10 postinfection, and as early as day 4 postinfection [47]. This latter finding revealed that the T‐bet+ ABCs originated from B cell precursors already present very early after infection. This was an important finding in its own right, which supported the idea that memory B cells can be detected in early GCs [48]. *Aicda* was also found to be constitutively expressed in T‐bet+ ABCs. In addition to these basic findings, the approach also provided a means of permanently marking the memory B cells in vivo (i.e., by administration of tamoxifen to the transgenic mice).

### 1.6 CD11c + T‐Bet+ Memory ABCs Can Function as Memory Stem Cells

**FIGURE 1:**
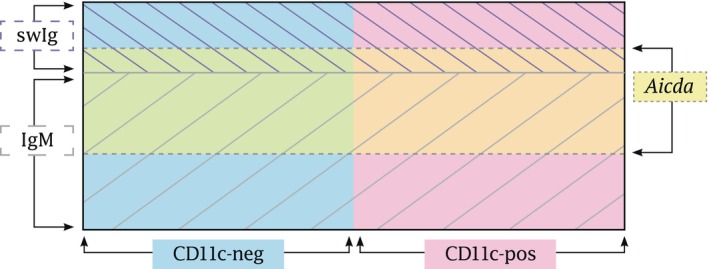
Heterogeneity of T‐bet + ABCs. A Venn diagram is shown that illustrates the populations of T‐bet+ memory ABCs detected in the spleens of *E. muris‐*infected mice. These include CD11c‐negative and ‐positive B cells; within these two major populations are cells that are switched and nonswitched (indicated with cross‐hatching), as well as cells that had expressed or lacked the enzyme AICDA (in green and orange). (Reproduced, with permission, from Kenderes et al. ref. [8]).

Although T‐bet+ ABCs were shown to formally function as memory B cells, the next objective was to characterize their differentiation following secondary infection, or antigen challenge. Using the inducible (AID‐Cre‐ER^T2^ × Rosa26 eYFP) F1 mice, it was possible to perform in‐depth analyses of the eYFP‐labeled IgM memory cells (i.e., T‐bet+ memory ABCs), as well as a population of B cells that had undergone class switching (swIg B cells), generated following *E. muris* infection [8]. The memory cells identified in this fashion (i.e., on the basis of their expression of *Aicda*), expressed T‐bet, the chemokine receptor CXCR3, CD11b, CD73, the costimulatory receptors CD80 and CD86, PD‐L2, CD95, the prosurvival BAFF‐receptor, the Ig class‐switch‐inducing receptor TACI, and the costimulatory molecule ICOS‐ligand, all cell surface markers identified in our previous studies of CD11c + T‐bet+ B cells, and T‐bet+ ABCs [6, 8, 49]. Using this approach, a significant population of CD11c‐negative T‐bet+ ABCs was identified [8]. These cells had not been identified in previous studies, which relied on CD11c as a marker for the T‐bet+ ABCs. Indeed, this latter work revealed that the T‐bet+ ABC population was more diverse than was first proposed and included several distinct subpopulations, some resembling memory cell populations that would likely be found in other infections or immunological contexts. Both CD11c‐postive and ‐negative, and AICDA‐positive and ‐negative subsets were detected. A Venn diagram of the memory cells identified in vivo has been reproduced in Figure 1. These studies revealed that T‐bet+ ABCs elicited during ehrlichial infection are functionally diverse. Moreover, it was concluded, on the basis of these studies, that T‐bet expression does not drive the development of any unique or novel memory B cell population. Rather, although T‐bet is the only marker that, to our knowledge, is uniformly expressed by the CD11c + B cells elicited during ehrlichial infection, the transcription factor performs a rather limited, although essential, function (see discussion below).

The inducible (AID‐Cre‐ER^T2^ × Rosa26 eYFP) F1 model system described above was also used to address the differentiation of T‐bet+ ABCs following challenge infection or immunization. This was accomplished by flow cytometric cell sorting and adoptive transfer of quiescent eYFP+ cells obtained from infected mice following tamoxifen administration [8]. The purified T‐bet+ ABCs were transferred to naive mice that were then challenged with OMP‐19 twelve days later (as indicated above, immunization was necessary because secondary *E. muris* is nonproductive in previously infected mice). Consistent with an amnestic response, a fraction of the transferred T‐bet+ ABCs underwent extensive differentiation following challenge. Remarkably, the donor T‐bet+ memory ABCs generated all known effector B cell linages, including spleen and bone marrow ASCs, IgM and swIg GC B cells, extrafollicular plasmablasts, and swIg memory cells; moreover, the T‐bet+ memory ABCs were maintained indefinitely, and were capable of responding to a secondary challenge (indicating their capacity to self‐renew, an essential property of memory B cells) [8]. Molecular characterization of B cell receptors from transferred T‐bet+ memory ABCs facilitated the construction of clonal lineage trees, independently confirming that these B cells were indeed multipotent, because the same BCR clone could be detected in several different populations of differentiated progeny. These studies reinforced the concept that T‐bet expression in B cells does not direct the differentiation of any particular B cell lineage; rather, the T‐bet+ ABCs formally function as memory stem cells. While the concept that memory lymphocytes are, by definition stem cells, that is, they can differentiate into multiple lineages, and self‐renew, was not novel, to our knowledge this was the first demonstration that memory B cells also embodied the property of stemness [50]. This presumably allows maximal flexibility in the secondary B cells/antibody response to antigenically‐related pathogens.

Using the adoptive transfer approach described, a role for CD11c expression in T‐bet+ ABC differentiation was also addressed. This was performed by independent transfer of CD11c‐negative and ‐positive eYFP+ donor T‐bet+ ABCs and challenge infection, using the same approach as described above. The two populations underwent very similar pathways of differentiation characteristic of unseparated memory cells [8]. Moreover, CD11c + and CD11c‐negative donor cells interconverted in vivo. Thus, ironically, although CD11c was the marker that facilitated the identification of T‐bet+ ABCs, no essential role(s) for the two subsets in memory B cell differentiation were identified, as CD11c‐negative and ‐positive T‐bet+ ABCs exhibited apparently identical responses. More likely, CD11c regulates cell migration and retention in lymphoid tissue under steady‐state conditions, or its functions are complemented by the related integrin, CD11b.

### 1.7 Pathways of T‐Bet+ Plasmablasts and T‐Bet+ ABC Development During Ehrlichiae Infection

**FIGURE 2:**
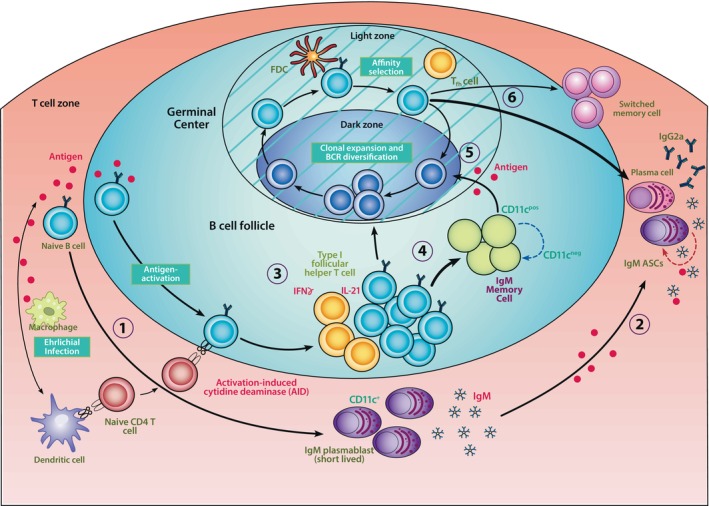
A model for the differentiation of T‐bet+ B cell‐derived plasmablasts and T‐bet+ ABCs in secondary lymphoid tissues during ehrlichial infection. The schematic describes the CD4 TI IgM+ T‐bet+ plasmablast response (1–2), and the generation and differentiation of TD memory T‐bet+ ABCs (3–6). Note that the two responses are temporally and anatomically distinct; the spleen T‐bet+ plasmablast response reaches a maxima on about day 18 postinfection and the T‐bet+ memory ABC response is fully developed at about 30 days postinfection. The latter response may occur primarily in the lymph nodes. The early spleen T‐bet+ plasmablast response (1) develops extrafollicularly, independent of GCs, and leads to the generation of short‐lived IgM+ T‐bet+ plasmablasts. Some of these T‐bet+ plasmablasts migrate to the bone marrow to become long‐lived IgM+ antibody‐secreting cells that provide long‐term immunity to ehrlichial infection (2). Later during the B cell response, CD4 T cell‐dependent T‐bet+ ABCs undergo differentiation, likely in B cell follicles (3). Help via IL‐21 and IFNγ is provided by Type I follicular helper T cells. Some of the T‐bet+ ABCs differentiate to CD11c + and −negative IgM memory cells, which undergo interconversion (4). Following reinfection or the introduction of antigen, the IgM+ T‐bet+ memory ABCs undergo clonal expansion and diversification, likely in GCs (5). The light and dark zones of the GCs are hatched to indicate that these structures may not be fully intact. This process leads to the generation of switched T‐bet+ memory ABCs, and some of these memory cells fully differentiate into IgG2a‐producing plasma cells.

The use of the ehrlichia infection model not only facilitated the discovery of T‐bet+ ABCs, but also led to studies of their development and differentiation. This work was aided by the use of the infection model, which, for reasons that are still uncertain, elicits an unusually large population of T‐bet+ ABCs. There is no reason to think that T‐bet+ ABCs are unusual or distinct from those generated in other infections, or in autoimmunity. Rather, as suggested above, it is likely that ehrlichial infection induces innate immune responses that favor differentiation of a very large TI spleen plasmablast response, and a smaller TD IgM and swIg memory response. Ironically, given the magnitude of the innate response, the ehrlichiae are not known to encode TLR ligands [51]. However, the bacteria elicit large inflammatory cytokine responses, especially the production of IFNγ [52]. IFNγ production is also partly responsible for driving large changes in hematopoiesis that accompany *E. muris* infection [53]. Although no evidence has been uncovered to suggest that altered hematopoiesis drives T‐bet+ ABC development, infection does drive a large expansion of Lin‐negative, Sca‐1+, cKit+ LSK cells in the bone marrow that may indirectly affect the generation of T‐bet+ ABCs [53]. Given the inflamed lymphoid environment induced by infection, unexpectedly, the inflammatory cytokine TNF appears to inhibit the expansion of GC B cells, T‐bet+ plasmablasts, and T‐bet+ ABCs, because these populations underwent expansion following TNF ablation [26]. One explanation for these findings is that early ehrlichial infection inhibits spleen GCs, and at the same time vastly enlarges the extrafollicular space [37], thereby providing a special environment for the development of T‐bet+ B cells. Indeed, T‐bet+ plasmablasts generated early in *E. muris* infection are preferentially found in the extrafollicular space in both the spleen and liver [37, 54]. Later during infection, the T‐bet+ ABCs appear to be follicularly‐derived. Splenic architecture is largely disorganized during their development, but following resolution of infection the follicles are largely restored [54]. Moreover, the lymph nodes, unlike the spleen, appear to remain intact during infection [37], possibly providing a site for the generation of TD T‐bet+ ABCs [54]. Regardless, major changes in the lymphoid environment are clearly not essential for the genesis of T‐bet+ ABCs, given that the B cells are now known to be generated in a plethora of viral infections that do not induce large changes in lymphoid architecture [15, 16, 55]. Alternatively, *E. muris* infection may drive the generation of T‐bet+ ABCs due to the type of CD4 T cell response it elicits. *E. muris* infection generates a large population of CD4 T cells that express PD1+, CXCR5+, and Bcl6 (although T‐bet+ ABCs develop normally when Bcl6 is deleted from T cells). However, T‐bet+ ABCs do not develop when T‐bet is eliminated from T cells. Indeed, *E. muris* infection generates a large number of T_H1_‐like cells capable of producing both IL‐21 and IFNγ. These data demonstrate the importance of T_H1_‐like help, and IFNγ, in the generation of T‐bet+ ABCs. Understanding the mechanisms that are responsible for generating very large T‐bet+ ABC responses in ehrlichia‐infected mice may facilitate the design of adjuvants specifically designed to generate robust protective T‐bet+ ABCs to other pathogens. Considering all these observations, it is proposed that two, perhaps independent, anatomically‐distinct pathways of T‐bet+ B cell development are utilized during ehrlichial infection. One is TI, and leads to the generation of IgM‐secreting T‐bet+ spleen and bone marrow plasmablasts. The other is TD, occurs partially or wholly in the lymph nodes, and leads to the generation of IgM+ and switched memory T‐bet+ ABCs. A model for T‐bet+ plasmablast and T‐bet+ ABC development and differentiation is shown and described in Figure 2. During ehrlichial infection, both pathways appear to provide protection to secondary infection, because mice lacking T‐bet+ ABCs or IgM are fully protected from *E. muris* infection [35, 52].

### 1.8 Role of T‐Bet in T‐Bet+ ABCs

Because T‐bet+ is a lineage‐defining transcription factor in T‐bet+ ABCs, an obvious question was whether the factor was essential for B cell development or function. This question was first addressed by Rubtsova and colleagues, who ablated T‐bet expression in CD19‐positive B cells in lupus‐prone mice. This was performed by using T‐bet floxed mice that had been crossed to CD19‐Cre recombinase‐expressing mice, thereby deleting T‐bet in all B cells [56]. Importantly, it was demonstrated that T‐bet deletion led to a reduction in lupus‐associated kidney pathology and mortality, concomitant with the reduction of the production of autoantibodies, in particular IgG2a [56]. This study confirmed that T‐bet was a major driver of B cell‐mediated autoimmunity during lupus. However, CD11c was still expressed in B cells in the gene‐targeted mice, suggesting that “T‐bet” B cells/ABCs do not require the transcription factor for their development. This observation has been confirmed in subsequent studies [57, 58].

We also addressed a possible role for the T‐bet transcription factor, using the ehrlichial infection model [52]. In our study, all B cells were targeted using (Mb1‐cre × T‐bet^fl/fl^) F1 mice (Mb1 also known as CD79a is a B cell‐specific factor known to be specifically expressed in all B cells). B cells lacking T‐bet developed normally, and were indistinguishable from wild‐type T‐bet+ ABCs with regard to their cell surface expression of CD11c, CD73, CD38, PD‐L2, and CD80. Instead, consistent with other studies, deletion of B cell‐specific T‐bet reduced the IgG2c response [52, 56, 57]. Thus, the primary and central role of T‐bet in B cells appears to be not in lineage specification, but rather, in Ig class switching to isotypes that provide protective immunity, or in other contexts, autoimmunity. This latter observation likely explains the importance of T‐bet+ ABCs in disease [4]. Although more recent studies have shown that another factor, ZEB2, and likely yet others, are important for the development and differentiation of T‐bet+ ABCs [59], T‐bet expression remains the definitive marker that delineates this important B cell population.

### 1.9 T‐Bet+ ABCs in Autoimmunity

The first description of ABCs in humans was from a study of patients with SLE [6]. That study, and others, described the cells in aged mice and humans [7, 60, 61, 62, 63]. Once identified, ABCs were eventually shown to be associated with a number of autoimmune diseases in humans and mice, including rheumatoid arthritis, Sjøgren's Syndrome, and SLE [13, 49, 64, 65]. As suggested above, a common role of T‐bet+ ABCs in these different autoimmune disease appears to be their production of IgG2a, an antibody isotype that has been described to be pathogenic in SLE [66, 67]. In SLE, T‐bet+ ABCs correlate with autoantibody production and disease severity, are responsive to TLR7 stimulation, and exhibit reduced expression of proinflammatory cytokines [68]. Moreover, reminiscent of our studies of early B cell responses following ehrlichia infection, it has been proposed that T‐bet+ ABCs can differentiate into plasmablasts that produce autoreactive antibodies [6, 49]. Importantly, experimental studies demonstrated that elimination of the T‐bet transcription factor (although not “T‐bet” B cells), ameliorated disease in lupus‐prone SLE1.2.3. mice (the mice carry three susceptibility loci known to contribute to disease) [56, 69]. These studies together suggested an important role for T‐bet+ ABCs not only in the etiology of SLE, but also other autoimmune diseases.

### 1.10 Molecular Characterization of T‐Bet+ ABCs: Differential Expression of the A2aR

Although initial work had focused on flow‐cytometric based phenotypic characterization of T‐bet+ ABCs during ehrlichial infection, a molecular analysis was also performed on these cells. Flow‐cytometrically sorted CD11c‐positive and ‐negative B cells were harvested after day 30 postinfection, and were analyzed by RNAseq. A summary of these findings were reported in Winslow et al. [41]. It was reassuring that CD11c and T‐bet were differentially expressed in the CD11c + (T‐bet+) B cells (47‐ and 7.8‐fold respectively, relative to CD11c‐negative B cells), validating the overall approach (note that although we later discovered CD11c‐negative T‐bet+ ABCs, these were likely under‐represented in the total CD11c‐negative B cell population that had been purified, and therefore did not appear to affect the analysis). The expression of a number of other genes encoding cell surface receptors that had previously been described in T‐bet+ ABCs, using flow cytometry, was confirmed. These included CD29, CXCR3, CD21, CD23, as well as several Fc receptors (including the inhibitory receptor FcγRIIa), the putative IgM receptor FcμR, TNF receptor 2, the IL‐2Rβ, IL‐10Rα, and TLR9. The functions of some of these factors remain to be defined in T‐bet+ ABCs, and is a fertile ground for further investigation. Other genes notably expressed at much higher frequencies relative to CD11c‐negative B cells were *S1pr5*, which encodes the sphingosine‐1‐phosphate receptor 5 (S1pR5; well‐known to regulate egress of B cells from lymphoid tissues), *Nod1*, which encodes the cytosolic innate receptor Nod1, and *Adora2a*, the gene encoding the G‐protein‐coupled adenosine receptor A2a (A2aR; expressed 10‐fold higher relative to CD11c‐negative B cells). The expression of *Adora2a* was of particular interest, because adenosine receptors have been studied at length in a number of physiological contexts, and many drugs have been developed to target these receptors in vivo. Moreover, a few studies have demonstrated that A2aR signaling can repress GC T_FH_ differentiation [70, 71]. Because adenosine receptors, in particular A2aR, are readily drug‐targeted, a possible role for the A2aR in T‐bet+ ABCs was addressed.

### 1.11 A Role for Adenosine Signaling in T‐Bet+ ABC Function During Ehrlichial Infection

Four adenosine receptors are encoded and expressed in the laboratory mouse (A1R, A2aR, A2bR, and A3R), and in humans [72]. The receptors are expressed on a variety of tissues, primarily in the nervous system, although they also have been reported to be expressed in leukocytes, endothelial cells, and neurons [73, 74, 75]. There has been significant clinical interest in compounds that target adenosine receptors, and both natural and synthetic antagonists and agonists are common. Indeed, several compounds have been developed that target these receptors, some in clinical use, for the treatment of a range of human diseases, including cancer [76, 77, 78]. Among these many agents, we focused on compounds that specifically targeted the A2aR.

Adenosine acts transiently; indeed, the half‐life of adenosine in vivo is 10 s [79]. Thus, adenosine acts at short distances in vivo. T‐bet+ ABCs presumably produce extracellular adenosine, as these B cells express on their surface CD73, CD39, and CD38, enzymes which are responsible for the production of extracellular adenosine, via the catabolism of ATP and NAD+ [74]. Indeed, CD73 was identified early as a signature surface marker on T‐bet+ ABCs in our early studies [1]. A role for adenosine in B cell signaling or function remains poorly understood and understudied, potentially due to a lack of tools, as antibody reagents and fluorescent agonists or antagonists have not been useful for detecting A2aR expression on B cells (unpublished data).

Among the many compounds and drugs that target adenosine include those that act as antagonists and agonists. Adenosine itself is typically not used clinically because of its very short half‐life. However, many antagonists and agonists have much longer half‐lives (some lasting days) [80]. A well‐known A2aR antagonist is caffeine, which inhibits adenosine signaling via all four adenosine receptors [81]. Agonists, which trigger receptor activity, include CGS‐21680, Binodenoson, NECA (5′‐*N*‐Ethylcarboxamidoadenosine) and others; one naturally occurring agonist, limonine, is found in citrus fruit [82, 83]. An agonist that is used clinically is Lexiscan. The latter agent is commonly used for physiological stress testing of cardiac patients that are unable to perform exercise‐induced stress testing [77, 84]. The agonist stimulates A2aR expressed on coronary smooth muscle, dilating coronary arteries and causing hyperemia [84]. Although Lexiscan targets A2aR, we utilized the agonist CGS‐21680 in most of our experiments, because a number of studies had reported use of this agonist in animal model experiments [70, 85, 86]. Indeed, experimental studies of mice that had been treated with the A2aR agonist CGS‐21680 demonstrated that the agonist inhibited GC B and T cell responses in vivo [70]. The authors of that study showed that the GC inhibition was due to the effects of the agonist on CD4 T cells, by impairing their differentiation into T_FH_ cells. While the role of A2aR signaling on T cells remains an important avenue of research, given our findings, we focused our studies primarily on B cell‐intrinsic functions of the A2aR.

Because the A2aR was differentially expressed at the mRNA level in T‐bet+ ABCs in the molecular analyses, a possible role for A2aR on T‐bet+ ABCs during ehrlichial infection was addressed. Initial studies utilized the well‐described A2aR antagonist istradefylline [87]. Administration of istradefylline (132 μg, every other day, from days 30–37 post‐ *E. muris* infection of C57BL/6 mice) failed to reveal any significant changes in the development or differentiation of T‐bet+ ABCs (unpublished data) [88]. Therefore, the approach was modified by using the A2aR agonist CGS‐21680; this agonist has been reported to bind A2aR with high affinity (although lower affinity interactions with the A1 and A3 receptors have been reported) [89]. Following treatment of C57BL/6 mice with CGS‐21680 (50 μg) on days 30–37 post‐ *E. muris* infection, a significant depletion of CD11c + T‐bet+ ABCs, but not of canonical CD11c‐negative B cells, was observed on day 37 postinfection [88]. Follow up studies revealed that T‐bet+ ABC depletion could be observed within as early as 24 h postadministration, indicating that the agonist acted rapidly. A role for other ARs in our model was not addressed, because only A2aR was found to be significantly upregulated in T‐bet+ ABCs; a role for other ARs cannot, however, be ruled out at this time.

No long‐term morbidity was detected in the agonist‐treated mice, which exhibited only a transient loss of movement. Moreover, no effect on bacterial clearance was observed [88]. Depletion of T‐bet+ ABCs did not lead to enhanced susceptibility to infection, likely due to protective pre‐existing antibodies, so this latter observation was not unexpected. Similar observations were obtained when the clinically‐approved agonist, Lexiscan, was used [88].

Because A2aRs are expressed on many different cells in vivo, any of which could have been the target of the administered agonists, it was next addressed whether the effect of CGS‐21680 on the T‐bet+ ABCs was due to its direct activity on the T‐bet+ ABCs. This was accomplished by administering the agonist to infected gene‐targeted (Mb1^cre/+^ × Adora2a^flox/flox^) F1 mice that lacked expression of A2aR only on B cells (although this approach targeted the A2aR on all Mb‐1+ B cells, high‐level A2aR had not been detected in non‐T‐bet+ B cells). Importantly, B cells were not targeted by CGS‐21680 in infected F1 B cell‐specific A2aR‐deficient mice [88]. This was a critical finding, demonstrated that the agonist had acted directly on the targeted T‐bet+ ABCs. Other studies demonstrated that the apparent loss of the B cells was not because they had egressed from the spleen; T‐bet+ B cells in other tissues, including LNs, bone marrow, and liver, were largely unaffected, for reasons that are not understood. One possibility is that T‐bet+ ABCs in other tissues do not express sufficient A2aR to be targeted.

These studies demonstrated not only that A2aR agonists can directly target T‐bet+ ABCs, but also suggested that the A2aR was not necessary for the short‐term maintenance or function of T‐bet+ ABCs (long‐term effects of targeting the A2aR on B cells was not addressed). Nevertheless, although the A2aR is likely nonessential, or is redundant, for normal T‐bet+ ABC function, nonphysiological triggering of the receptor, using an agonist, can nonetheless lead to T‐bet+ ABC death, likely via apoptosis. It is also possible that the A2aR does not signal strongly on T‐bet+ ABCs under steady state conditions, as extracellular adenosine is tightly regulated [90].

### 1.12 Drug Targeting T‐Bet+ ABCs in Autoimmune Mice

The association of T‐bet+ ABCs with a number of autoimmune diseases in mice and humans suggested that specific targeting could provide an opportunity to eliminate or curtail the activity of T‐bet+ ABCs, using agonist‐mediated A2aR targeting. Lupus‐prone mice were utilized because (1) several available strains of mice have been reported that model SLE, and (2) gene‐targeted depletion of T‐bet in SLE1.2.3. mice had been shown to reduce symptoms of SLE [56]. Yet other studies reported that agonist treatment of lupus‐prone mice reduced disease severity, although the mechanism whereby this occurred was unknown [91].

To address possible effects of agonist treatment of autoimmune‐prone mice, the well‐established MRL/lpr model for SLE was utilized [92]. MRL/lpr mice typically begin to exhibit symptoms of lupus within 8 weeks of age and begin to succumb by 20 weeks of age [93]. Disease symptoms include production of anti‐chromatin and ‐RNA antibodies, glomerulonephritis and interstitial nephritis, lymphadenopathy, dermatitis, and lymphoproliferation. The MRL/lpr mice were treated with the agonist CGS‐21680 twice weekly, starting at 8 weeks of age (vehicle‐treated mice were used as controls), and the mice were monitored thereafter. Similar to data from studies of ehrlichia‐infected mice, a marked decrease in T‐bet+ ABCs in the agonist‐treated mice was observed at 20 weeks of age [88]. Some depletion was also observed on CXCR5+ PD1+ CD4+ T cells, but the population of FoxP3+ T regulatory cells, which are thought to play role in SLE pathogenesis, was unaffected. These observations were not model‐dependent, as a similar reduction in T‐bet+ ABCs were made using SLE1.2.3. mice [88]. These data revealed that it was possible to target T‐bet+ ABCs in autoimmune mice.

We next assessed whether the targeting of T‐bet+ ABCs was accompanied by a reduction in disease in the agonist‐treated MRL/lpr mice. A number of differences in the treatment group (at 20 weeks of age) were observed, including (1) a reduction in anti‐RNA and dsDNA‐specific antibodies, (2) a reduction in splenomegaly, lymphadenopathy, and reduced kidney pathology, and (3) a modest improvement in survival times [88]. Moreover, interstitial kidney nephritis was markedly reduced. These studies demonstrated that A2aR agonist administration could be used to treat autoimmune disease in laboratory mice. Indeed, disease could also be ameliorated when treatment was delayed (in these later study's treatment was commenced at 20 weeks of age, well after disease onset). These studies revealed that agonist treatment may be effective when used therapeutically. This work also suggested that pharmacological elimination of T‐bet+ ABCs may be effective for the treatment not only of SLE, but of other autoimmune diseases, such as Sjøngren's disease, RA, Celiac's disease, Crohn's disease, and others, where T‐bet+ ABCs are considered to be major effector cells [12, 13, 64, 94]. Agonist treatment, where animal models for these diseases are available, could be readily tested.

### 1.13 Therapeutic Use of A2aR Agonists in Humans

As indicated above, increased expression of the A2aR on lymphocytes has been documented in patients with SLE, which suggests that targeting of the A2aR on T‐bet+ ABCs may be a viable approach to treat autoimmune diseases in patients [95]. This could be accomplished using A2aR agonists, or other reagents that target the receptor. Agonist antibodies directed against the A2aR have not yet been reported, although these may be possible to generate. Limited studies were performed by treating blood T‐bet+ ABCs in vitro with CGS‐21680, and a significant reduction of T‐bet+ ABCs was observed in some human samples following 24 h of CGS‐21680‐treatment (unpublished data). There are a number of reasons to suggest why agonist treatment may not be successful in vitro, so the preliminary findings are very encouraging. These studies were performed using blood from healthy humans, as it is well‐known that many humans, especially older individuals, carry T‐bet+ ABCs in peripheral blood [63]. It isn't known if the T‐bet+ ABCs in patients and in healthy humans are functionally or phenotypically equivalent, but this is likely the case. Critical differences in T‐bet+ ABCs from normal humans and patients more likely exist in BCR specificity for autoantigens. This work also suggests that preliminary clinical studies regarding cell targeting need not necessarily be conducted in patients, although ultimately clinical evaluations will be necessary.

As indicated above, at least one adenosine agonist (Lexiscan) is FDA‐approved, and is commonly used for myocardial imaging. Some of the side effects from the use of this agonist are headache, dizziness, chest pain, and blood–brain barrier disruption [96, 97]. However, these are well‐tolerated, and generally resolve within about 15 min; side‐effects can also be reversed using an adenosine antagonist such as caffeine [96]. Clearly, nonlymphocytes will also be agonist targets in humans, and this off‐site targeting may not be tolerated, but it eventually may be possible to limit transient side‐effects.

## 2 Future Directions

The findings suggest that additional studies in healthy humans and patients are warranted. Human studies are well‐within the capabilities of current technologies. A particular advantage of the use of A2aR agonists for the treatment of diseases such as SLE is that the approach targets T‐bet+ ABCs directly. This presents a significant advantage over current clinical approaches, which target all B cells. Although CD4 T cells play an important role in lupus, most likely by providing B cell help, efforts to target CD4 T cells may be less effective than targeting of the B cells directly responsible for generating pathogenic antibodies. Nevertheless, a full evaluation of the effects of agonist treatment in humans will by necessity involve monitoring its effects on T cells. Indeed, a loss of CD4 T cells was observed in MRL/lpr mice following early, but not delayed, administration of CGS‐21680 [88]. Importantly, the parameters required for optimal and effective agonist treatment have yet to be established humans. Therapeutic treatment for autoimmunity will likely require repeated agonist administrations, perhaps for indefinite periods. Although frequency of administration required to limit disease in humans is not yet known, modest and transient side effects may be well‐tolerated in otherwise healthy patients. Nor is the dosage required for moderating T‐bet+ ABCs in humans known. For example, it is possible that frequent low‐dose treatment of patients with agonists could be effective, if administered over an extended period; this is possible due to the lengthy course of many autoimmune diseases.

Alternatively, if side‐effects are a major barrier to agonist therapy, it may be feasible to generate B cell‐specific A2aR agonists that avoid or limit off‐target effects. This may be possible using multi‐functional regents, for example, agonist‐coupled monoclonal antibodies that target an invariant B cell surface receptor and the A2aR, or bifunctional antibodies that target A2aR and an invariant B cell surface protein. Cell‐type specific targeting is a goal of many drug designers, so advances in this area of research should provide guidance for the use of adenosine agonists in patients [98].

An additional concern is that targeting T‐bet+ ABCs via the A2aR may also deplete/impact protective T‐bet+ ABCs (and perhaps some CD4 T cells) responsible for protecting humans from infections, although this has yet to be tested. As was demonstrated in the studies of infected mice, many T‐bet+ B cells differentiate into memory cells or memory stem cells. Although antibodies are well‐known to provide long‐term protection, it is possible that long‐term T‐bet+ ABC depletion would eventually compromise immunity to pathogens. Whether this is a concern will depend on a cost–benefit analysis that compares benefits to limiting autoimmunity to loss of vaccine efficacy, or protection against infection. One possible approach that may ameliorate this concern, however, given our knowledge of T‐bet+ ABCs, is that these B cells differentiate under conditions of Type I immunity/inflammation (i.e., IFNγ is a primary driver of T‐bet+ ABC responses) [7, 99]. Immunizations that are designed to generate Type II immunity, for example, by using novel adjuvants, may facilitate the generation of nonpathological protective memory B cells that resist targeting by A2aR agonists [100]. Together, this work demonstrates the utility of basic research and animal models, as well as the study of underrepresented pathogens, for the discovery of novel mechanisms and treatment modalities for human diseases.

## Conflicts of Interest

The authors declare the following competing interests: G.M.W. and R.C.L. are inventors listed on a patent pending for the use of A2A receptor agonists in the therapeutic depletion of CD11c + T‐bet+ B cells in diseases mediated by these B cells. Applicant: SUNY Research Foundation, Application Serial Number: PCT/US2019/045624.

## Data Availability

Data sharing is not applicable to this article as no new data were created or analyzed in this study.
